# Perception and awareness towards malaria vaccine policy implementation in Nigeria by health policy actors

**DOI:** 10.1186/s12936-023-04536-z

**Published:** 2023-03-29

**Authors:** Adaugo Nnaji, Macide Artac Ozdal

**Affiliations:** 1 Institute of Graduate Studies and Research, European University of Lefke, Northern Cyprus, TR-10 Mersin, Turkey; 2 Faculty of Health Sciences, Department of Health Management, European University of Lefke, Northern Cyprus, TR-10 Mersin, Turkey

**Keywords:** Vaccines, Malaria control, Policy

## Abstract

**Background:**

This study aimed to assess the perception and awareness of malaria vaccine policy implementation among health policy actors in Nigeria.

**Methods:**

A descriptive study was conducted to assess the opinions and perceptions of policy actors on the implementation of a vaccination programme against malaria in Nigeria. Descriptive statistics were carried out to study the characteristics of the population and the univariate analysis of the responses to questions presented to the participants. Multinomial logistic regression was conducted to evaluate the association between demographic characteristics and the responses.

**Results:**

The study revealed that malaria vaccine awareness was poor, with only 48.9% of the policy actors having previous knowledge of the malaria vaccine. The majority of participants (67.8%) declared that they were aware of the importance of vaccine policy in efforts to manage disease transmission. As the number of years of work experience of the participants increased, the odds of being more likely to be aware of the malaria vaccine increased [OR 2.491 (1.183–5.250), p value < 0.05].

**Conclusion:**

It is recommended that policy-makers develop methods of educating populations, increase awareness of the acceptability of the vaccine and ensure that an affordable malaria vaccine programme is implemented in the population.

## Background

The malaria burden in Nigeria accounts for 25% of global cases. The causes include climate, high transmission potential, socioeconomic development, overstretched health care systems, and displaced populations [1, 2]. The disease is frequently treated in Nigeria through self-prescription as well as the utilization of nearby herbs, spiritualist/traditional priests and health facilities/hospitals [3, 4]. Additionally, normal control measures incorporate the utilization of medication (prophylaxis), insect sprays (coil and sprays), insecticide-treated nets (ITNs), and window and door nets [5, 6]. Artemisinin-based combination therapy (ACT) is used as recommended treatment for uncomplicated malaria in Africa. In Nigeria, artemether-lumefantrine is used as an essential drug because of its efficacy in the treatment of malaria [7].

In Nigeria, malaria kills approximately 400,000 people per year, mostly young children. The malaria vaccine known as Mosquirix, RTS,S/AS01, or simply RTS,S is the first vaccine proven to offer partial protection against malaria [8, 9]. The World Health Organization (WHO) recommends that in the context of comprehensive malaria control, the RTS.S malaria vaccine should be used for the prevention of *Plasmodium falciparum* malaria in children living in regions with moderate to high transmission as defined by the WHO. The RTS, S malaria vaccine is provided in a schedule of four doses for children from 5 months of age for the reduction of malaria disease and burden [10].

To eliminate malaria in Nigeria, there must be sincere and sustained commitment by the government, policy-makers and citizens to eliminate malaria in Nigeria [11]. It is essential to improve existing malaria control measures and targeted interventions to reduce the large burden of the disease and reduce the direct or indirect health care cost for the treatment and management of the disease [12, 13]. The development of an anti-malarial vaccine is important for regional malaria elimination and future eradication efforts. Malaria vaccine policy is a global effort, and the vaccine has already been applied to a large number of children in Ghana, Kenya, and Malawi [13–15].

There have been efforts to evaluate the effectiveness, cost-effectiveness, efficacy and safety impact of malaria vaccines implemented in the three regions of Ghana, Kenya, and Malawi [15, 16]. The pilot programme coordinated by the WHO indicates that the vaccine is successful in terms of safety and feasibility [18]. Evaluation studies investigate ways of implementing vaccine policies more effectively and efficiently by assessing the dosage and schedule of vaccines administered. This will provide lessons for the implementation of further effective and cost-effective malaria vaccine programmes [16–18].

The Nigerian vaccine policy was proposed in 2021. The purpose of the implementation of this vaccine policy was to address the objective and goals of accomplishing accessibility, independence and vaccine security in the country [19]. It is believed that the improvement of this policy will supplement the existing vaccine policy and assist in the enhancement of vaccine-preventable infections in Nigeria. Evidence shows that policy actors have sufficient knowledge of the benefits of the vaccine and its impact on recipients, which is a reduction in the severity of malaria in Nigeria. It is suggested that to eliminate malaria in Nigeria, there must be an absolute resolution by the government to explore all available options to upscale existing malaria control measures. For strategies or policies to be successful, it is essential to consider certain factors [20]. The governance of a nation plays a significant role in policy implementation. Furthermore, the cycle and stages a policy undergoes determine its success, although bureaucracy prompts delays in the execution of policy [21]. Nigeria has three systems of government: executive, legislative and judiciary bills are initiated by the executive arm and then introduced to the legislature where deliberations are conducted; it takes time before a consensus is reached and then passed into law [22]. The judiciary reviews bills and determines the constitutionality of legislative, executive and bureaucratic actions and policies [23]. The judiciary guarantees that each government action aligns with the goal and objective of the laws, which is to improve the wellbeing of the public [24]. For malaria vaccines to be added to the existing malaria control measures, the policy process should be void of unnecessary bureaucracy [25]. The malaria vaccine is a new control measure that should be targeted by the Nigerian government.

The current study aims to assess the knowledge and perceptions of policy actors on the implementation, safety and financing of a malaria vaccine policy in Nigeria. The study will provide important implications for the successful implementation of a malaria vaccine policy in Nigeria.

## Methods

### Study subjects and methods

The study was designed as a descriptive study, and quantitative data were collected using a questionnaire. The questionnaire was conducted among officials employed in institutional bodies concerned with the prevention and control of malaria in Nigeria. Informed consent was obtained from all participants before they completed the questionnaires. The study methodology was approved by the Scientific Research and Publication Ethical Committee of the European University of Lefke. Permission was also granted from all the institutions in which the participants were employed.

### Population and sampling technique

The population target for the data collection for the research was officials acting as health policy actors who were involved directly in the eradication of malaria. The target organizations were nongovernmental organizations (NGOs), federal government organizations, associates/societies, and academia (public health lecturers). A non-probability sampling method, namely, purposive sampling, was used in this study since the total population number of policy actors employed in the target organizations was not apparent. Depending on the area of their interest and relevance of their interests in the current study, 90 individuals were approached, and all agreed to participate in the study.

### Method of data collection

Data for the study were collected during June 2021 through a questionnaire with a 5-point Likert scale ranging from 1 (strongly agree) to 5 (strongly disagree). The survey was conducted face to face among officials in institutional bodies working toward the prevention and control of malaria in Nigeria. The questionnaire consisted of a written informed consent form. The questionnaire was adapted from the research questions related to the study aims and objective [26]. The relevant questions were adapted to the present study, and questions that were not relevant were omitted.

The questions were grouped into sections that were used to work toward the aims of the study, namely, awareness of the malaria vaccine and malaria vaccine policy; perceptions of policy actors on the implementation of malaria vaccine policy in Nigeria; and willingness to access and pay for the malaria vaccine in the case of implementation of a malaria vaccine programme.

### Data analysis

The collected data were analysed using the Statistical Package for Social Science research (SPSS). Validity and reliability tests were conducted with 15 individuals in the target group. The participants of the reliability test were selected from all the organizations involved as target institutions. The reliability was tested using the Cronbach’s alpha measure, which was determined to be 0.852, greater than the threshold of 0.7 used for assessing the reliability of the questionnaires.

Descriptive statistics were then conducted to determine the sociodemographic characteristics of the respondents and the frequency of the responses made by the participants to the questions in the survey. Inferential analyses were conducted using multinomial regression to assess the associations between cofactors such as the number of years of work experience, gender, age of the participants, and profession to determine the confounding factors that affected awareness of the malaria vaccine and perceptions of the implementation of malaria vaccination measures in Nigeria. The goodness of fit of the model including the demographic cofactors was studied based on Pearson and deviance residuals for the final decision on inclusion of the cofactors in each model. Those that did not fit well into the model were not included.

## Results

Table 1 presents the characteristics of the study participants based on gender, age, level of education, years of working experience in their current job, position, type of institute and profession. The study population consisted of 90 respondents, of whom 51.5% were male and 48.9% were female. A total of 46.7% of the participants were aged between 29 and 39, and 28.9% were aged between 18 and 28. There was only one respondent who was a primary school graduate, while 16.7% were secondary school graduates, 28.9% were high school graduates, and 25.6% were university graduates. Twenty-three out of 90 participants (27.8%) had postgraduate education. The majority of participants (42.2%) had less than 5 years of experience in the field, while 24.4% of the participants had more than 10 years of experience. A total of 81.1% of participants (73 participants) were in a staff position in the organization in which they were employed. Half of the participants were employed in government organizations, while 20% of them were academics employed in a university. Forty-two respondents representing 46.7% of the study population acted as health personnel, and the rest were not health personnel.

**Table 1:** Characteristics of the study participants who acted as policy-makers employed in governmental organizations, nongovernmental organizations or university organizations, and nongovernmental organizations or universities

Characteristics	N	%
Gender		
Male	46	51.5
Female	44	48.9
Age of respondent		
18–28	26	28.9
29–39	42	46.7
40–50	13	14.4
51 and above	19	8.9
Level of education		
Secondary	16	17.8
High School	26	28.9
University	25	25.6
Post-graduate	23	27.8
Years of work experience		
Less than 5 years	38	42.2
5–10 years	30	33.3
More than 10 years	22	24.4
Type of institute		
Government parastatal	45	50.0
NGO	27	30.0
University	18	20.0
Type of profession		
Management	17	18.9
Staff	73	81.1
Total	90	100

Table 2 illustrates the awareness of malaria and the malaria vaccine among the respondents. It was observed that 46.7% of respondents were not aware of the existence of the malaria vaccine, while approximately half of the respondents were aware of the malaria vaccine (48.9%). More than half (67.8%) of the respondents indicated that they thought the malaria vaccine would be important for controlling malaria in Nigeria in response to the question, “*Do you think the malaria vaccine is a good addition to the control/eradication policy and program in Nigeria*?” The majority of respondents, with 80.0% responses, stated that they agreed that malaria episodes lead to low productivity and that the vaccine would help to curb this.

**Table 2:** Awareness of malaria and malaria vaccine prospects

Responses	Frequency	Percentage(%)
Do you have any knowledge of the malaria vaccine?		
No	42	46.7
Yes	44	48.9
I don’t know	4	4.4
Malaria episodes lead to loss of economic productivity		
No	15	16.7
Yes	73	80
I don’t know	2	3.3
Will the malaria vaccine be a good addition to the control/eradication policy and program in Nigeria?		
No	25	28.9
Yes	60	67.8
I don’t know	5	5.6

Table 3 presents the responses from the policy actors on their perception of the malaria vaccine. The majority of the respondents (86.7%) agreed that the malaria vaccine would reduce frequent hospital admissions, 85.7% agreed that the malaria vaccine would save money on reoccurring treatment, 14.0% were neutral about their response, and 1.1% of respondents disagreed. Approximately 81.1% strongly agreed that the malaria vaccine policy, if implemented, would reduce severe malaria anemia by 6 in 10. A total of 86.7% strongly agreed that the malaria vaccine would prevent 3 in 10 cases of severe malaria. Approximately 73.2% agreed that the vaccine could prevent 4 out of 10 malaria infections.

**Table 3:** Perceptions of malaria vaccines by policy actors

Responses	Frequency	Percentage (%)
Malaria vaccine will reduce frequent hospital admissions		
Strongly agree	63	65.0
Agree	23	34.0
Neutral	4	1.0
Disagree	0	0.0
Strongly disagree	0	0.0
Malaria vaccine will help save money spent on reoccurring treatment		
Strongly agree	41	45.0
Agree	37	40.0
Neutral	10	14.0
Disagree	2	1.0
Strongly disagree	0	0.0
Malaria vaccine policy, if implemented, will reduce 6 in 10 cases of severe malaria anemia		
Strongly agree	34	37.8
Agree	40	44.0
Neutral	14	11.0
Disagree	2	2.2
Strongly disagree	0	0.0
Malaria vaccine will prevent 3 in 10 cases of severe malaria		
Strongly agree	41	45.6
Agree	37	41.1
Neutral	9	10.0
Disagree	2	2.2
Strongly disagree	1	1.1
Malaria vaccine can prevent 4 out of 10 malaria infections		
Strongly agree	34	37.4
Agree	40	44.4
Neutral	14	15.6
Disagree	2	2.2
Strongly disagree	0	0.0

Table 4 illustrates the response of policy actors to their perceptions of malaria control policy measures. A total of 65.6% of respondents did not agree that the existing malaria treatment measures were sufficient in the eradication process. A total of 27.8% of participants responded that they did not think that the existing measures were sufficient in controlling malaria. The policy actors responded to the question about their perceptions on the easy implementation of the malaria vaccine policy; approximately half of the respondents thought that the malaria vaccine policy would be implemented easily, while 33.3% declared that they did not think that the malaria vaccine policy would be implemented easily.

**Table 4:** Perception of policy actors about malaria control policy measures

Responses	Frequency	Percentage
Are the existing malaria treatment measures sufficient in the eradication process?		
No	59	65.6
Yes	25	27.8
I don’t know	6	6.7
Will malaria vaccination be implemented easily as a policy?		
No	30	33.3
Yes	46	51.1
I don’t know	14	15.6

Table 5 illustrates that 74.4% of participants strongly agreed that there would be wide acceptance by the population, while 1.1% strongly disagreed with the wide acceptance of the malaria vaccine. Among the policy-makers, 55.1% strongly agreed that everyone should receive the malaria vaccine, 22.1% agreed, 15.6% were neutral, 5.6% disagreed, and 1.1% strongly disagreed. Table 5 indicates that 17.8% of policy actors thought that persons would be asked to pay for the vaccine, 77.8% did not agree with people having to pay as they believed it would be freely received when made available, and 4.4% of respondents did not know how the delivery process would take place.

**Table 5:** Thoughts of policy actors on willingness to accept and to pay for malaria vaccine

Responses	Frequency	Percentage (%)
Will the populace have wide acceptance of the vaccine?		
Strongly agree	63	70.0
Agree	23	25.6
Neutral	4	4.4
Disagree	0	0.0
Strongly disagree	0	0.0
Should everyone receive the malaria vaccine?		
Strongly agree	41	45.6
Agree	37	41.1
Neutral	10	11.1
Disagree	2	2.2
Strongly disagree	0	0.0
Will people be asked to pay for the malaria vaccine?		
Strongly agree	34	37.8
Agree	40	44.4
Neutral	14	15.6
Disagree	2	2.2
Strongly disagree	0	0.0

Table 6 presents the results of the multinomial regression analysis that was performed to examine the impact of demographic factors on awareness of malaria vaccine policy and perceptions of malaria control measures by policy actors. It was observed that as the number of years of work experience increased, policy actors were more likely to be aware of the existence of the malaria vaccine [OR 2.491 (1.183–5.250), p value < 0.05]. The older the respondents were, the significantly more likely they were to respond that they did not know whether the malaria vaccine would be implemented easily in Nigeria [OR 3.042 (1.231–7.516), p value < 0.05].

**Table 6:** The impact of demographic characteristics on awareness of malaria vaccine policy and perceptions of malaria control measures by policy actors: multinomial regression analysis results

	Work experience OR (95% CI)	Type of institute OR (95% CI)	Gender OR (95% CI)	Age OR (95% CI)	Profession OR (95% CI)
Do you have any knowledge of the malaria vaccine?					
No^#^	0.00	0.00	0.00	0.00	0.00
Yes	2.491(1.183–5.250)*	0.680(0.377–1.226)	1.756(0.676–4.560)	0.779(0.428–1.418)	1.488(0.583–3.794)
I don’t know	9.179(1.077–78.256)	0.185(0.019–1.805)	8.080(0.566–115.366)	0.379(0.063–2.261)	0.589(0.045–7.675)
Malaria episodes lead to loss of economic productivity. Will the vaccine be helpful in curbing this?					
No^#^	0.00	0.00	0.00	0.00	0.00
Yes	0.00	1.836(0.247–13.653)	0.00	3.896(0.393–38.657)	5.032(0.337–75.046)
I don’t know	0.00	2.198(0.322–15.024)	0.00	5.181(0.556–48.278)	2.607(0.207–32.813)
Do you think the malaria vaccine is a good addition to the control/eradication policy and program in Nigeria?					
No^#^	0.00	0.00	0.00	0.00	0.00
Yes	1.234(0.593–2.566)	1.041(0.563–1.923)	1.357(0.502–3.673)	0.885(0.483–1.623)	0.491(0.179–1.347)
I don’t know	0.965(0.193–4.836)	0.804(0.222–2.905)	1.768(0.227–13.769)	0.751(0.190–2.973)	0.789(0.102–6.136)
Are the existing malaria treatment measures sufficient in the eradication process?					
No^#^	0.00	0.00	0.00	0.00	0.00
Yes	0.717(0.337–1.525)	0.736(0.388–1.397)	1.589(0.584–4.320)	0.946(0.507–1.767)	1.002(0.370–2.717)
I don’t know	1.479(0.335–6.532)	0.468(0.188–2.229)	8.754(0.831–92.168)	0.941(0.288–3.072)	2.422(0.353–16.620)
Will malaria vaccination be easily implemented as a policy?					
No^#^	0.00	0.00	0.00	0.00	0.00
Yes	0.918(0.430–1.958)	1.233(0.663–2.294)	0.819(0.304–2.202)	1.571(0.774–3.189)	0.682(0.257–1.809)
I don’t know	0.572(0.194–1.693)	1.837(0.750–4.499)	3.089(0.693–13.778)	3.042(1.231–7.516)*	1.233(0.282–5.389)

^*^p value < 0.05

^#^Reference group

## Discussion

### Main findings

The findings of this study provide insight into policy actors’ knowledge and awareness of the malaria vaccine available in countries such as Ghana, Malawi and Kenya and their perceptions of the adoption of a malaria vaccine policy in Nigeria. The study sheds light on how the malaria control measures already in existence could be further improved. Malaria control measures practiced by the Nigerian government consist only of insecticide-treated bed nets and ACT [8], while other nations in Africa have implemented malaria vaccine programmes. This study, which aimed to examine the knowledge and awareness of the malaria vaccine and the perceptions of the implementation of vaccine policy in Nigeria by participants who act as policy actors in the country, included approximately half male and half female participants, and the majority of participants were aged between 29 and 39 years. The majority of participants had less than 5 years of experience in the field and were high school, university or postgraduate students. When awareness of the malaria vaccine was studied, only approximately half (48.9%) of the participants stated that they had heard about the malaria vaccine. Eighty percent of the participants agreed that malaria episodes leading to loss of economic productivity could be addressed by a malaria vaccine policy, and 67.8% of participants were aware of the importance of including a malaria vaccine policy in the existing malaria control measures in Nigeria. The study further examined the perceptions of policy actors on the malaria vaccine and found that over 80% of the participants strongly agreed or agreed that the malaria vaccine has the potential to control malaria cases and reduce disease burden in the population. When the study respondents were asked, “*Are the existing malaria treatment measures sufficient in the eradication process*?”, half of the participants did not agree that the existing malaria treatment and control measures were sufficient in the process of eradicating malaria in the Nigerian population, 33.3% of participants did not think that the existing measures in Nigeria were helpful in controlling malaria in the country, and 16.6% participants had no idea about the potential benefits of the malaria vaccine for malaria control. Regarding the questions on the willingness to adopt the malaria vaccine in Nigeria, 70.0% of participants strongly agreed that a malaria vaccine program would be strongly accepted by the population. The study further indicated that the responses of the participants changed based on demographic characteristics. Those with more years of experience were more likely to have knowledge and awareness of the malaria vaccine implemented in other African countries but not in Nigeria [6]. The perception of the adoption and implementation of a malaria policy was low, with only approximately half of the study population thinking that malaria vaccines would be adopted easily in Nigeria and approximately 20% of the study population stating that they were not sure if malaria vaccine implementation would be straightforward. The older the respondents were, the more likely they were to state that they did not know whether the vaccination policy would be easily adopted. This may be because more experience in the effort to address health problems in the population leads to reduced confidence in the statement that a new policy on the malaria vaccine will be easily adopted in Nigeria [27].

### Explanation of findings

This study showed that awareness of the malaria vaccine among policy actors was low. This finding is consistent with the findings of another study that assessed awareness of the malaria vaccine among health care providers in Southeast Africa, where 48.6% of 500 participants stated that they were aware of a malaria vaccine [27]. In another study performed on knowledge and attitude toward the malaria vaccine among mothers in Nigeria, awareness of the malaria vaccine was 30%, which is lower than the level of awareness among policy actors [28]. Studies on knowledge, attitudes and perceptions toward polio immunization indicated that education is vital in enhancing awareness of public health problems and the attainment of health interventions and policies, such as the malaria vaccination policy [29, 30]. The low awareness of the malaria vaccine among health care staff in the present study indicates that formal education may not be sufficient to raise awareness of health interventions to address health problems. Previous studies have emphasized that there is a need for consistent communication and continuous education of the population on important public health issues to attain a high level of awareness in the whole population [31, 32]. Awareness of malaria and malaria vaccine policies was also shown to be associated with religion, household income and marital status in a trial conducted in Ghana. The present study showed that the awareness level of health policy actors in the development of public health policies is associated with the number of years of work experience. A higher number of years spent in the field may contribute to greater awareness of the adverse impacts of malaria on the population and the intention to search for and gain knowledge on public health interventions, such as malaria vaccines [33].

The policy actors involved in the current study highly agreed that when the malaria vaccine policy is implemented, the population will be asked to pay for the vaccine. Previous studies have shown that not being able to afford the cost of public health interventions and limited health expenditure may reduce compliance with interventions to address malaria [34]. Interventions with no cost have been shown to be more successful in achieving coverage. Studies on the willingness to pay for malaria vaccines show that target populations have reduced willingness to pay for the vaccine, and acceptance would be higher if it is provided for free [27, 35].

Although this study suggests that the Nigerian population will be accepting of receiving the malaria vaccine, there are a number of factors associated with the acceptance of the vaccine, such as cultural practices, efficacy, beliefs and the existence of adverse impacts of the vaccine [36–38]. Asmare showed that mothers may not be willing to vaccinate their children against malaria since they believe that the vaccine may harm their children, it may be expensive and their husbands will not allow their children to be vaccinated [35]. It is, therefore, important to increase knowledge through the education and support of the populace in accepting and increasing awareness of the potential benefits and adverse impacts of being vaccinated against malaria. The promotion of vaccine policies relies strongly on the Nigerian government in terms of providing education to improve knowledge on the benefits of vaccination. Therefore, the government should develop methods for improving knowledge on the benefits of malaria vaccines to enhance their acceptance and coverage in the population.

## Limitations

The responses to the study were limited because of the COVID-19 outbreak that started at the time of data collection. The lockdowns because of the pandemic affected communication between respondents and the time allocated for completing the questionnaire. The study employed a quantitative approach in collecting data to explore awareness and perceptions of malaria vaccine programmes, which potentially leads to the loss of valuable views and thoughts from the study participants that could be collected using a qualitative research approach [39].

## Conclusions

There must be a sincere and sustained commitment by the government, policy-makers and citizens to eliminate malaria in Nigeria through consistent implementation of control measures, such as vector control interventions, effective diagnosis of cases and effective treatment of identified cases. The specified existing control measures must be supported by effective and cost-effective prevention interventions including malaria vaccines. Although the current study showed a commitment by the policy actors toward the importance of the development of a vaccine policy intervention, there are still questions regarding the poor knowledge of the malaria vaccine and the affordability of this intervention in Nigeria. In the process of developing a malaria vaccine intervention, it is important to provide education to the population to increase their knowledge of the malaria vaccine and its benefits for enhanced acceptance. It is also important to conduct research and find methods for the cost-effective application of malaria vaccine policies in populations.

## Data Availability

Materials used for data collection can be accessed upon request.
